# The characteristics of patients with hypermagnesemia who underwent emergency hemodialysis

**DOI:** 10.1002/ams2.334

**Published:** 2018-02-21

**Authors:** Mana Nishikawa, Noriaki Shimada, Motoko Kanzaki, Tetsunori Ikegami, Toshio Fukuoka, Masaki Fukushima, Kenichiro Asano

**Affiliations:** ^1^ Department of Nephrology Kurashiki Central Hospital Kurashiki Japan; ^2^ Department of Emergency Medicine Kurashiki Central Hospital Kurashiki Japan; ^3^ Department of General Medicine Kurashiki Central Hospital Kurashiki Japan; ^4^ Department of Internal Medicine Shigei Research Institute Hospital Okayama Japan

**Keywords:** Acute kidney injury, elderly, magnesium oxide

## Abstract

**Aim:**

This study aimed to clarify the characteristics of patients who presented with severe hypermagnesemia and subsequently underwent emergency hemodialysis.

**Methods:**

We investigated the age, gender, complications, clinical symptoms, causal drugs, electrocardiogram findings, and laboratory data of 15 patients.

**Results:**

Magnesium oxide had been administered in all cases and 14 patients were over 65 years old. The male : female ratio was 6:9. Chief complaints included a disturbance of consciousness, hypotension, bradycardia, and respiratory failure. The median serum magnesium value before hemodialysis was 6.0 (3.7–18.6) mg/dL. The daily dosage of magnesium oxide was ≤ 2.0 g in 12 cases. The median serum creatinine value before hemodialysis was 5.39 (0.54–10.29) mg/dL. However, in two cases, the creatinine value was not elevated. Complications of acute kidney injury exacerbated the hypermagnesemia in nine cases.

**Conclusions:**

We recommend that the serum magnesium value should be measured in older patients who are taking magnesium oxide and are showing signs and symptoms of a disturbance of consciousness, hypotension, bradycardia, and respiratory failure of an uncertain etiology, even if the serum creatinine value is not elevated or the dosage of magnesium oxide is within recommended levels.

## Introduction

Maintaining magnesium (Mg) in the body in just the right proportion is essential for life.^1^ Mg plays a role as a cofactor in multiple enzymatic reactions such as energy metabolism and DNA and protein synthesis, and Mg is related to the regulation of ion channels.^1^ In hypermagnesemia, because Mg acts as a calcium channel blocker,^2^, ^3^ the blood vessels tend to dilate, which leads to lower blood pressure levels, and it also causes prolonged QT interval, increased QRS duration, and bradycardia on electrocardiograms, and cardiac arrest.^3^ Furthermore, patients with hypermagnesemia present a disturbance of consciousness.^4^ In addition, hypermagnesemia may cause respiratory failure^2^ due to respiratory muscle paralysis because hypermagnesemia may inhibit the release of acetylcholine at the nerve synaptic terminal.^5^ Hypermagnesemia is easily overlooked because of its non‐specific symptoms. Mild hypermagnesemia causes general malaise, such as nausea and vomiting, whereas serious hypermagnesemia can result in death.^6^

Although reports exist on hypermagnesemia requiring urgent hemodialysis (HD),^6^ there are few reports on the characteristics of such cases. Therefore, we retrospectively analyzed cases of hypermagnesemia requiring emergency HD at our hospital and investigated the characteristics of these patients.

## Methods

**Figure 1:**
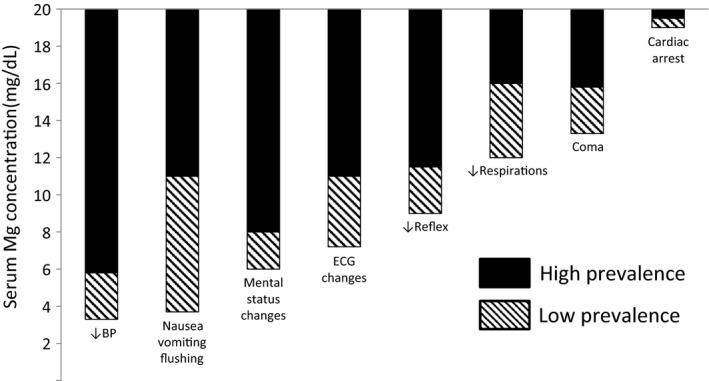
Serum magnesium (Mg) concentration and the clinical manifestations. Serious adverse symptoms occur in almost all patients in the higher ranges of serum Mg concentration (solid columns), whereas the symptoms may be observed in some patients in the lower range of serum Mg concentration (hatched columns). Reproduced from Mordes and Wacker 2 with permission from The American Society for Pharmacology and Experimental Therapeutics. To convert mEq/L to mg/dL, multiply the values by 1.2.^37^ BP, blood pressure; ECG, electrocardiogram.

We included in the study 15 outpatients and inpatients who underwent emergency HD due to hypermagnesemia at our hospital during the 24‐year period from January 1992 to December 2015. Children and maintenance dialysis patients were excluded. The indications to carry out emergency HD for hypermagnesemia at our hospital included fatal clinical symptoms due to hypermagnesemia and a decision that HD was necessary for the removal of Mg. The decision was made by multiple doctors, such as emergency physicians, emergency department doctors, and nephrology doctors. The serum Mg values at which clinical symptoms may appear are approximately: ≥3.6 mg/dL for lowered blood pressure, nausea, vomiting, and bradycardia; ≥6.0 mg/dL for changes in an individual's mental state and a disturbance of consciousness; ≥10.8 mg/dL for respiratory failure and coma; and ≥16.8 mg/dL for cardiac arrest (Fig. 1).^2^ In our study, we analyzed the subjects’ age, gender, clinical symptoms, complications, electrocardiogram findings, oral medicine before HD, the serum Mg value and the serum creatinine (Cre) value before HD, the serum Cre value before discharge or death, and estimated glomerular filtration rate (eGFR). In cases of death, the cause of death was analyzed. The reference range of the serum Mg value at our hospital ranged from 2.0 to 2.6 mg/dL, which was measured by the chelate (xylidyl blue) method. The reference range of the serum Cre value was 0.60–1.10 mg/dL for men and 0.45–0.80 mg/dL for women, which was measured using enzyme methods. For eGFR, the estimation formula^7^ described in the “CKD Clinical Practice Guide 2012” from the Japanese Society of Nephrology was used. In the clinical practice guideline for acute kidney injury (AKI), KDIGO,^8^ an increase in the serum Cre value before HD of ≥1.5‐fold from the serum Cre base value was defined as AKI when setting the serum Cre values before discharge or death as the base value. Patients were classified according to the increase in serum Cre value: Stage 1, increased by 1.5 to 1.9‐fold; Stage 2, increased by 2.0 to 2.9‐fold; and Stage 3, increased by ≥3.0‐fold. This study was carried out with the approval of the ethics committee of Kurashiki Central Hospital.

## Results

The median age of the subjects was 78 years (range, 59–94 years), with 14 elderly people aged ≥65 years (Table 1). There were six men and nine women, 11 outpatients and four inpatients. The median serum Mg value before HD was 6.0 (3.7–18.6) mg/dL. The clinical symptoms observed before HD included 10 cases of a disturbance of consciousness, five cases with lowered blood pressure, five cases of bradycardia, and two cases of respiratory failure. There were only two cases of flushing. In addition, complications included nine cases of chronic kidney disease (CKD), six cases of hyperkalemia, one case of untreated hypothyroidism, and one case of ileus. There were no complications of inflammatory bowel disease or Addison's disease. Oral medicine included two cases of vitamin D preparations, four cases of angiotensin receptor antagonists (ARB), two cases of non‐steroidal anti‐inflammatory drugs (NSAIDs), and no cases of treatment with angiotensin converting enzyme inhibitors (ACE‐I). An electrocardiogram before HD was undertaken in 13 cases, and the findings included PR interval prolongation in seven cases, prolonged QT interval in six cases, bradycardia in four cases, and atrioventricular block in three cases, with no observed increased QRS duration. Two patients died during hospitalization; the causes of death were blood disease and an aortic aneurysm rupture. There were no deaths due to hypermagnesemia.

**Table 1:** Characteristics of patients with hypermagnesemia who underwent emergency hemodialysis (*n* = 15)

Age, years	Gender	Dose of oral MgO (mg/day)	Serum Mg before HD (mg/dL)	Serum Cre before HD (mg/dL)	Serum Cre before discharge/death (mg/dL)	eGFR before discharge/death (mL/min/1.73 m^2^)	NSAIDs	ARB	AKI stage	Factors related to Mg metabolism	Background factor
68	F	1980	15.1	0.54	0.26	186.4	Y	N	2		DM, HT, Parkinson's disease, pneumonia
74	M	2000	3.7	4.04	0.85	67.4	N	N	3		DM, HT, acute glomerulonephritis, pneumonia
76	F	1980	8.1	0.84	0.48	92.3	N	N	1	Vit D	
78	F	2000	7.1	7.13	1.43	27.8	Y	N	3	Vit D	HT, rheumatoid arthritis,
										CKD	urinary tract infection
79	F	Dose unknown	5.3	10.29	2.95^a^	12.5	N	Y	3	CKD	HT, congestive heart failure, abdominal aortic aneurysm (cause of death)
82	F	1500 + Magnesium citrate 34 g + Magnesium aluminometasilicate 1.2 g	18.6	0.90	0.33	136.1	N	N	2	Ileus	HT, depression
83	F	2000	7.0	7.06	1.55	25.0	N	N	3	Hypothyroidism CKD	HT, dementia, neurogenic bladder
92	M	990	5.3	10.18	3.60	13.0	N	Y	2	CKD	HT
94	M	1980	4.5	6.00	3.28	14.4	N	Y	1	CKD	HT
59	F	1320	6.8	0.62	0.60	77.8	N	N			DM, catheter‐related bloodstream infection
69	M	1980	4.7	1.56	2.07^a^	26.0	N	N			DM, HT, leukemia (cause of death)
76	F	2000	6.0	9.93	8.17	4.2	N	N		CKD	DM, HT
77	M	3000	7.3	4.31	3.49	14.2	N	N		CKD	DM, HT, pneumonia
85	M	3000	5.6	5.39	5.54	8.3	N	N		CKD	DM, HT, congestive heart failure
87	F	990	6.0	5.45	4.10	8.5	N	Y		CKD	HT

Hypothyroidism and Addison's disease are risk factors of hypermagnesemia, because these diseases increase tubular magnesium (Mg) reabsorption.^38^.

AKI, acute kidney injury; ARB, angiotensin II receptor blocker; CKD, chronic kidney disease; Cre, creatinine; DM, diabetes mellitus; eGFR, estimated glomerular filtration rate; F, female; HD, hemodialysis; HT, hypertension; M, male; MgO, magnesium oxide; N, no; NSAIDs, non‐steroidal anti‐inflammatory drugs; Vit D, vitamin D; Y, yes

a Patient death during admission.

### Renal function

The median serum Cre value before HD was 5.39 (0.54–10.29) mg/dL. Nine cases involved AKI (Table 1), including two Stage 1 cases, three Stage 2 cases, and four Stage 3 cases, according to the AKI classification. In two cases, the serum Cre value before HD did not increase and was below the upper limit of the reference range. In all cases, HD was able to be discontinued after the emergency HD. The median serum Cre value before discharge or death was 2.07 (0.26–8.17) mg/dL, and the median eGFR was 25.0 (4.2–186.4) mL/min/1.73 m^2^.

### Oral magnesium oxide (MgO)

**Figure 2:**
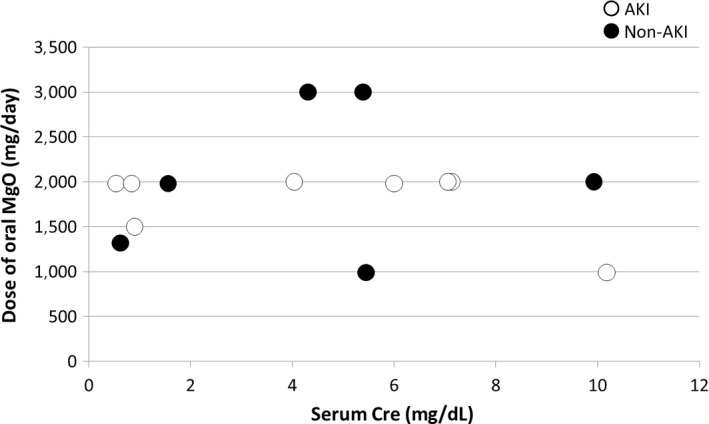
Dose of oral magnesium oxide (MgO) and serum creatinine (Cre) values in 15 patients with hypermagnesemia, before they underwent emergency hemodialysis. One patient was taking an unknown dose of MgO. AKI, acute kidney injury.

Oral MgO was administered in all cases. In 14 cases, excluding one with an unknown dose, the maximum daily dose of oral MgO up to 3 days before HD was ≤1.0 g in two cases, 1.1–2.0 g in 10 cases, and ≥2.1 g in two cases. There were 12 cases in which the dose of oral MgO was 2.0 g/day or less, which is the regular dose (≤1.2 g/day in terms of Mg content). In addition, one patient was taking stomachics and digestives, including 1.2 g magnesium aluminometasilicate (approximately 0.1 g in terms of Mg content) and 34 g magnesium citrate (approximately 2.7 g in terms of Mg content) as a pretreatment for colonoscopy. Figure 2 shows the dose of oral MgO and the serum Cre values before HD. In two cases, there was no elevation of the serum Cre value before HD and the patients were given regular doses of oral MgO.

**Figure 3:**
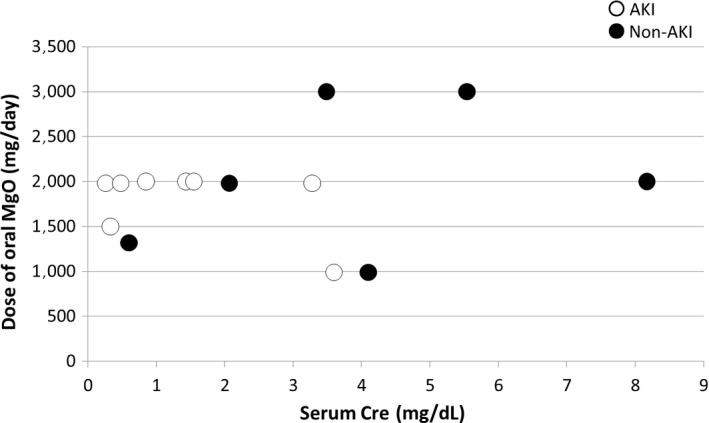
Dose of oral magnesium oxide (MgO) before emergency hemodialysis and serum creatinine (Cre) values before discharge or death in 15 patients with hypermagnesemia who underwent emergency hemodialysis. One patient was taking an unknown dose of MgO. AKI, acute kidney injury.

Figure 3 shows the dose of oral MgO before HD and the serum Cre values before discharge or death. In this study, the serum Cre value before discharge or death is defined as the base renal function. In seven cases, the serum Cre base values were ≤2.00 mg/dL and regular doses of oral MgO were given. Six of these were elderly people, all of whom were had with AKI and had hypermagnesemia.

## Discussion

At our hospital, 93% of those who required emergency HD due to hypermagnesemia were elderly people aged ≥65 years. Clinical symptoms before HD included a disturbance of consciousness (67%), lowered blood pressure (33%), bradycardia (33%), and respiratory failure (13%). Oral MgO was taken in all cases. Of the patients whose dosage was known, 86% were taking ≤2.0 g/day, which is the regular dose. The serum Cre value before HD was within the reference range in two cases (13%). The incidence of association with AKI was 60%. Based on these findings, it is possible that hypermagnesemia may have been caused by AKI and other reasons when elderly people taking oral MgO visited the emergency room with symptoms including a disturbance of consciousness, lowered blood pressure, bradycardia, and respiratory failure. We believe that it is necessary to measure the serum Mg value regardless of the dosage of oral MgO or the serum Cre value.

Renal function, which is the main excretion route of Mg,^9^ is important when considering the cause of hypermagnesemia. Glomerular filtration rate (GFR) that tends to lead to hypermagnesemia is ≤30 mL/min.^9^, ^10^ It is reported that eGFR of <30 mL/min/1.73 m^2^ in elderly people in Japan who take MgO orally, is associated with a risk of hypermagnesemia.^11^ In our study, for the group in which eGFR before HD was <30 mL/min/1.73 m^2^, it might not have been possible to excrete the ingested Mg, including oral MgO, sufficiently due to a decline in renal function. In addition, elderly people accounted for 93% of all patients in this study. As the renal function declines with age, old age is a potential risk factor for hypermagnesemia.^12^ According to the “Guidelines of safe medical therapy for the elderly” from the Japan Geriatrics Society,^13^ it is also strongly recommended to monitor the serum Mg value when elderly people with a declining renal function are treated with oral MgO.

Serum Cre values are generally used for estimating renal function. However, in this study, fatal hypermagnesemia occurred even though the serum Cre value was not higher than the reference range. The reason for this is that in elderly people, especially in women, the serum Cre values are less likely to rise despite a reduced renal function, as Cre production is less due to the lowering of muscle mass. eGFR can be used to estimate renal function taking age and gender into account.^7^ However, as GFR is estimated using the standardized body surface area of 1.73 m^2^, an overestimation of eGFR occurs if the physique is small.^14^ In this study, eGFR before discharge or death of two patients was ≥100 mL/min/1.73 m^2^, which we considered as overestimated. Even if the renal function worsens due to AKI, the serum Cre value does not rise immediately. We consider that it takes several days until the serum Cre value can be regarded as a remarkable rise.^15^ Based on these findings, we believe that there is a limit to ruling out renal dysfunction even if the serum Cre values or eGFR are within the reference range, based on a single blood sample taken in the emergency room.

**Figure 4:**
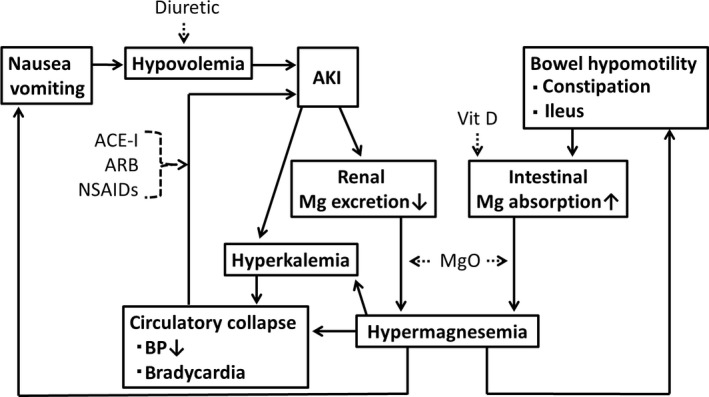
Vicious cycle of hypermagnesemia. Hypermagnesemia induces acute kidney injury (AKI) because of hypotension, hypovolemia, or bradycardia. AKI decreases renal magnesium (Mg) excretion and exacerbates hypermagnesemia. Hypermagnesemia also decreases renal potassium excretion and induces hyperkalemia.^3^ In addition, hypermagnesemia induces bowel hypomotility. The prolonged contact of Mg with the mucosa enhances intestinal Mg absorption. Because of increased Mg intake, magnesium oxide (MgO) could be a risk factor for hypermagnesemia. ACE‐I, angiotensin‐converting enzyme inhibitor; ARB, angiotensin II receptor blocker; BP, blood pressure; NSAIDs, non‐steroidal anti‐inflammatory drugs; Vit D, vitamin D.

Furthermore, even if the dosage of oral MgO is the regular dose, hypermagnesemia could potentially occur depending on concomitant drugs and complications. There are two vicious circles of hypermagnesemia (Fig. 4). One is caused by AKI. If AKI occurs for some reason, such as dehydration following nausea and loss of appetite due to mild hypermagnesemia, the excretion of Mg from the kidney decreases. Furthermore, the circulatory dynamics may deteriorate due to bradycardia or lowered blood pressure due to hypermagnesemia, which may also result in AKI.^16^, ^17^ Hypermagnesemia is a risk factor for AKI.^18^ Various drugs can also affect this vicious circle. Among patients taking ACE‐I, ARB, or NSAIDs, there is a clinical condition called normotensive ischemic AKI that is caused by mild dehydration or mildly lowered blood pressure.^19^ At the time of dehydration and lowered blood pressure, the kidney is capable of automatic adjustment to maintain the glomerular filtration pressure by dilating the afferent arteriole through prostaglandin and contracting the efferent arteriole through angiotensin II.^19^ However, it is impossible to properly dilate the afferent arteriole in patients taking NSAIDs, which reduce prostaglandins. Furthermore, patients taking ACE‐I or ARB cannot contract the efferent arteriole properly. Therefore, in patients taking ACE‐I, ARB, or NSAIDs, mildly lowered blood pressure at a systolic blood pressure of approximately 100–115 mmHg needs to be carefully monitored so as not to lead to AKI.^19^ Elderly people are at risk for developing AKI,^20^ including normotensive ischemic AKI.^19^ In this study, even among elderly people without a significant increase in the serum Cre base values, at ≤2.00 mg/dL, and taking regular doses of MgO, all patients had AKI as a complication and presented with hypermagnesemia.

The second vicious cycle of hypermagnesemia is through the intestinal tract. Magnesium that is ingested orally, including MgO, is mainly absorbed in the small intestine, particularly in the proximal portions.^9^ Although MgO is often used as a laxative, the prolonged contact of the mucosa with Mg due to constipation enhances the intestinal absorption of Mg.^17^ Inflammatory bowel disease enhances the intestinal absorption of Mg.^21^ When the serum Mg value increases, the movement of the intestinal tract further decreases due to the neuromuscular blocking action of Mg. As a result, the time when Mg remains in the intestinal tract is prolonged, the amount of Mg absorbed from the intestinal tract increases, and hypermagnesemia thus becomes exacerbated.^17^ Vitamin D preparations given as a therapeutic agent for osteoporosis also have an accelerating effect on Mg absorption in the jejunum.^9^ As the incidence of the complications of osteoporosis^22^ and constipation is high^23^ in elderly people, it is necessary to pay attention to this vicious circle.

Hypermagnesemia is easily overlooked because, even if the dose of oral MgO is regular and there is no rise in the serum Cre value, it may nevertheless sometimes still occur with non‐specific symptoms. Flushing, as a relatively characteristic clinical symptom of hypermagnesemia,^2^ was only observed in two cases in this study. As a method to identify severe hypermagnesemia patients, some reports indicate that the serum Mg values should be screened among patients with abnormal respiration, circulation, and consciousness in emergency rooms.^6^ Others suggest that hypermagnesemia should be considered if an elderly person has lethargy, hyporeflexia, paralytic ileus, hypotension, bradycardia, or respiratory depression, regardless of the oral administration of Mg.^12^ If elderly people have clinical symptoms such as a disturbance of consciousness, lowered blood pressure, bradycardia, and respiratory failure, along with a history of oral administration of MgO, regardless of the dosage of oral MgO or the serum Cre value, it is desirable to measure the serum Mg value. The main causes of death involving hypermagnesemia include intestinal ischemia, intestinal necrosis, and sepsis due to a decrease in blood pressure^6^, ^12^, ^24^, ^25^ with the serum Mg value ranging from 7.4 to 23.6 mg/dL,^6^, ^12^, ^24^, ^25^, ^26^, ^27^, ^28^, ^29^, ^30^, ^31^ ≤10.0 mg/dL in one case, 10.1–15.0 mg/dL in five cases, 15.1–20.0 mg/dL in two cases, and ≥20.1 mg/dL in two cases. If it can be confirmed that respiratory failure and circulatory insufficiency are caused by severe hypermagnesemia by measuring the serum Mg value, Mg can be quickly removed by HD,^3^ which may enable lives to be saved. In order to prevent mild hypermagnesemia from advancing to severe hypermagnesemia, attention should be paid to avoid AKI. In addition, treatment with concomitant medications should be confirmed and MgO or any commercial drugs, such as bitterns, including Mg^32^ should be reduced or discontinued. When interpreting the serum Mg value, it is necessary to note that the serum Mg values associated with the appearance of clinical symptoms may vary (Fig. 1). Although the Mg value measured in the laboratory is often the total serum Mg value, the total serum Mg value does not always reflect the total amount of Mg in the body. Active Mg in the living body is only ionized Mg. As a result, the total serum Mg value and clinical symptoms may not always be closely associated.^3^ Furthermore, similar to hyperkalemia,^33^ clinical symptoms of hypermagnesemia may be manifested due to the higher rate of rise in serum Mg other than the actual value of the serum Mg level.

This study was limited by the fact that the renal function before hypermagnesemia is unknown, and the original incidence of CKD could not be clearly evaluated.

Oral MgO is an excellent laxative which is inexpensive and whose long‐term use is usually well tolerated. Oral MgO is used by many patients (approximately 45 million people annually in Japan).^34^ However, the risk of fatal hypermagnesemia due to the oral administration of MgO has become common in recent years.^6^, ^34^ In addition, some reports indicate that hypermagnesemia has a negative effect on prognosis during hospitalization.^35^, ^36^ The prognosis of elderly people who receive oral MgO may be improved if care is taken to detect fatal hypermagnesemia and if the development of severe hypermagnesemia due to AKI is prevented.

## Conclusion

When elderly people taking oral MgO visit the emergency room with clinical symptoms including a disturbance of consciousness, lowered blood pressure, bradycardia, and respiratory failure, regardless of the dosage of oral MgO or the serum Cre value, there is a possibility that such individuals may be suffering from hypermagnesemia, and it is therefore necessary to measure the serum Mg value.

## Disclosure

Approval of the research protocol: This study was carried out with the approval of the ethics committee of Kurashiki Central Hospital (No.2151).

Informed consent: N/A.Registry and registration no. of the study/trial: N/A.Animal studies: N/A.

Conflict of Interest: None declared.
